# Current status of ECMO for massive pulmonary embolism

**DOI:** 10.3389/fcvm.2023.1298686

**Published:** 2023-12-21

**Authors:** Mark G. Davies, Joseph P. Hart

**Affiliations:** ^1^ Center for Quality, Effectiveness, and Outcomes in Cardiovascular Diseases, Houston, TX, United States; ^2^ Department of Vascular/Endovascular Surgery, Ascension Health, Waco, TX, United States; ^3^ Division of Vascular Surgery, Medical College of Wisconsin, Milwaukee, WI, United States

**Keywords:** ECMO, massive pulmonary embolism, outcomes, guidelines, cardiogenic shock, cardiac arrest

## Abstract

Massive pulmonary embolism (MPE) carries significant 30-day mortality and is characterized by acute right ventricular failure, hypotension, and hypoxia, leading to cardiovascular collapse and cardiac arrest. Given the continued high mortality associated with MPE, there has been ongoing interest in utilizing extracorporeal membrane oxygenation (ECMO) to provide oxygenation support to improve hypoxia and offload the right ventricular (RV) pressure in the belief that rapid reduction of hypoxia and RV pressure will improve outcomes. Two modalities can be employed: Veno-arterial-ECMO is a reliable process to decrease RV overload and improve RV function, thus allowing for hemodynamic stability and restoration of tissue oxygenation. Veno-venous ECMO can support oxygenation but is not designed to help circulation. Several societal guidelines now suggest using ECMO in MPE with interventional therapy. There are three strategies for ECMO utilization in MPE: bridge to definitive interventional therapy, sole therapy, and recovery after interventional treatment. The use of ECMO in MPE has been associated with lower mortality in registry reviews, but there has been no significant difference in outcomes between patients treated with and without ECMO in meta-analyses. Considerable heterogeneity in studies is a significant weakness of the available literature. Applying ECMO is also associated with substantial multisystem morbidity due to a systemic inflammatory response, hemorrhagic stroke, renal dysfunction, and bleeding, which must be factored into the outcomes. The application of ECMO in MPE should be combined with an aggressive pulmonary interventional program and should strictly adhere to the current selection criteria.

## Introduction

The development of pulmonary embolism continues to be a leading cause of preventable cardiovascular mortality and morbidity (1). Large-volume emboli within the pulmonary arterial tree result in sudden onset shock and cardiac arrest. This cohort accounts for approximately 5% of patients diagnosed with acute pulmonary embolism (PE) (2). Overall, patients who carry a diagnosis of PE have a 30-day mortality of 1%–2% if they remain hemodynamically stable, but the 30-day mortality increases to 10%–25% for hemodynamically unstable patients, and in those patients who have a cardiac arrest their 30-day mortality exceeds 50% (3–7). Deployment of Extra-Corporal Membrane Oxygenation (ECMO) in these high-risk hemodynamically unstable patients offers an oxygenation support strategy to improve hypoxia and an offloading strategy for right heart circulation, both contributing to the shock that develops in massive pulmonary embolism (MPE) (8, 9) This narrative review examines the current state of ECMO in critically ill patients with MPE.

## Experimental models

Experimental models have shown that no circulatory changes are observed until the cross-sectional area of the pulmonary vasculature is reduced by over 50% by pulmonary emboli. In response to increased pulmonary precapillary resistance, pulmonary arterial pressure and right ventricular systolic pressures rise. The ensuing hypoxemia and acute development of pulmonary hypertension induce the hyperactivation of the sympathetic response within the pulmonary vasculature, aggravating pulmonary artery spasm and further reducing pulmonary arterial blood flow induced by the emboli (10–13). Eventually, the increased pressure leads to the right ventricle (RV) dilatation with a rise in its end-diastolic pressure and a decrease in coronary flow. However, in comparison to the response seen in humans, the left ventricle (LV) in animal models is generally unaffected (14). Gurewich et al. demonstrated that the release of biogenic amines from platelets, triggered by thrombin, plays a significant part in the physiological response to pulmonary thromboembolism (15). The increase in pulmonary artery pressure and right ventricular wall tension leads to the release of B-type natriuretic peptide (BNP), leading to different degrees of increase in the concentration of BNP (both active BNP and inactive NT-proBNP) in the blood. When myocardial injury occurs, cardiac troponin is released. In addition, heart-type fatty acid binding protein (h-FABP), a soluble protein in the cytoplasm of cardiomyocytes, is released and quickly enters the blood when myocardial cells are damaged.

The degree of pulmonary occlusion rate is directly related to D-dimer and inversely associated with fibrinogen levels. In a canine model, pericardial constraint has been shown to contribute to hemodynamic deterioration during acute right ventricular pressure loading (16). In models of PE, there are significant changes in lung gene expression within 2 h of the index event, with upregulation of multiple pathways related to inflammation, immune disease, pulmonary disease, and cardiovascular disease. There is elevated expression of the chemokine genes CXCL1, CXCL2, CXCL3, and CCL2 (17). The increase in inflammatory genes allows new inflammatory markers to be reported: neutrophil–lymphocyte ratio (NLR), platelet–lymphocyte ratio (PLR), and lymphocyte–monocyte ratio (LMR). A PE also induces significant changes in microparticle characteristics, which develop a prothrombotic phenotype, further exacerbating the veno-occlusive process within the pulmonary vasculature (18). While, these changes in inflammatory and coagulation responses will allow for recovery of the pulmonary vasculature and parenchyma over time but the presence of an ECMO circuit results in additional inflammatory cascades that exacerbates the effects of the PE.

Using a rat model of veno-arterial (VA) and veno-venous (VV) ECMO, Cho et al. (19) reported that granulocytes are initially activated in both ECMO modalities, and this phenomenon does not normalize until three days after decannulation. VA-ECMO induces an initial reduction in monocyte and natural killer cells, and their levels were restored within three days of decannulation. The authors noted a significant decrease in B-lymphocytes, helper T-lymphocytes, and cytotoxic T-cells in VA-ECMO, but these cellular changes were not observed in VV-ECMO. Kjærgaard et al. developed a porcine model of MPE (20) where the investigators injected numerous thrombi into the right atrium of 18 pigs, which traveled into the pulmonary vasculature to simulate MPE, and the pigs were placed on full cardiopulmonary support. Once the MPE was induced and the animals were supported on the pump, one of three interventions was performed: normothermia, hypothermia, or tissue–plasminogen activator therapy. The study found that VA-ECMO can rescue pigs with MPE by allowing time for physiological compensation without a significant change in clot burden detected. This study offers experimental support for the use of VA-ECMO in MPE.

## Pathophysiology

The presence of an acute pulmonary embolus leads to detrimental changes in hemodynamic parameters within the pulmonary circulation, interferes with pulmonary gas exchange, and changes lung mechanical capacity (8, 21–23). Sixty-three percent of patients present with severe hypoxemia (PaO_2_ < 70 mm Hg) as a result of acute disruption of pulmonary physiology and consequent changes in gas exchange (21). While hypoxia contributes to the pathophysiology of pulmonary embolism, most early deaths result from acute RV pressure overload and subsequent RV failure (24). The shock seen in PE results from a rapid increase in pulmonary vascular resistance due to emboli entering and obstructing the pulmonary arterial vascular bed. Once lodged in the vascular bed, embolic material creates a mechanical obstruction. It induces an indirect increase in resistance through hypoxic and acidotic-induced vasoconstriction and triggers the release of vasoactive mediators from pulmonary artery endothelial and smooth muscle cells. This abrupt increase in pulmonary vascular resistance increases RV afterload, which produces RV dilation and myocardial dysfunction. The dysfunctional and dilated RV additionally impacts LV filling and significantly decreases LV preload, manifesting in decreased cardiac output, systemic hypotension, and, ultimately, cardiogenic shock.

## Classification

The American Heart Association (AHA) provided the original classification of three distinct pulmonary embolism events based on their respective anatomic and physiological findings: “Minor” to “Sub-massive” to “Massive” PE (25). This concept has been advanced by a classification system adopted by the European Society of Cardiology (ESC), which focuses on the associated risk of mortality associated with an acute PE event: “high,” “intermediate,” and “low” (26). Hemodynamic instability is defined as a systolic blood pressure <90 mmHg, hypotension requiring vasopressor support, or a decrement of the systolic blood pressure >40 mmHg for >15 min, or requiring inotropic support), pulselessness, or persistent profound bradycardia (25, 27, 28). Patients with MPE present with cardiac arrest, obstructive shock, or persistent hypotension (Table 1).

**Table 1:** Classification of massive pulmonary embolism.

Society	Category	Criteria
American Heart Association	Massive	Hemodynamic instability manifested by one of the following
		Systolic blood pressure < 90 mmHg for >15 min
		Requirement of inotropes
		Signs of shock
		Decrease from baseline Systolic blood pressure (BP) > 40 mmHg
		Cardiac arrest
		Significant symptom manifestation
		Hypotension
		Tissue hypoperfusion
		Hypoxemia.
European Society of Cardiology	High risk	Cardiovascular shock or persistent hypotension manifested by one of the following
		Cardiac Arrest with a need for cardiopulmonary resuscitation
		Obstructive shock with Systolic BP < 90 mmHg or vasopressors required to achieve a BP ≥90 mmHg despite adequate filling status and-organ hypoperfusion (altered mental status; cold, clammy skin; oliguria/anuria; increased serum lactate)
		Persistent hypotension manifested by Systolic BP < 90 mmHg or systolic BP drop ≥40 mmHg, lasting longer than 15 min and not caused by new-onset arrhythmia, hypovolaemia, or sepsis

## Current guidelines and ECMO

Both the American College of Chest Physicians (ACCP) and the European Society of Cardiology (ESC) initially developed evidence-based guidance and recommendations for the therapy of MPE (26, 29). The recommended therapeutic options for patients with MPE currently encompass surgical embolectomy (30), systemic thrombolysis (31), and catheter-based techniques (32). The most recent 2019 AHA scientific statement on MPE suggests that patients requiring therapeutic escalation through surgical embolectomy, catheter-directed thrombolysis, or systemic thrombolysis may be supported by ECMO, while these therapies are administered (33). In the same year, an updated set of ESC guidelines recommended open surgical embolectomy (Class I recommendation, Level C) or percutaneous catheter-based intervention (Class IIa recommendation, Level C) in patients with MPE at high risk of bleeding. The updated guidelines now propose that ECMO may be considered in conjunction with these interventions in those suffering refractory cardiogenic shock or cardiac arrest (Class IIb recommendation, Level C) if it is performed at a center of excellence with the necessary expertise and resources and the patient meets criteria for ECMO placement (27). To allow ECMO care standardization, the Extracorporeal Life Support Organization (ELSO) in 2021 published interim guidelines on deploying veno-arterial ECMO in adult patients with cardiovascular collapse (34).

## ECMO therapy

Extra-Corporeal Membrane Oxygenation (ECMO) is a form of partial cardiopulmonary bypass, which can be used for short-term support of respiratory and cardiac function in critically ill patients who are in a cardiopulmonary crisis (35). The primary purpose of ECMO, in all settings, is to successfully exchange oxygen and carbon dioxide from the blood using mechanic.al means in patients unable to accomplish this physiologically due to cardiopulmonary compromise (35). Hill reported the first successful human adult case of ECMO in 1972 (36), and Davies reported the first successful use of ECMO as a temporary support in massive pulmonary embolism outside the operating room in 1995 (37).

ECMO can be deployed in two circuits: veno-arterial perfusion or veno-venous perfusion. The veno-arterial ECMO circuits (VA-ECMO) are intended to take deoxygenated blood from a central vein or the right atrium, pass it through an oxygenator, and then return the oxygenated blood into the body by way of a central or a peripheral artery (35). This form of ECMO partially supports the cardiac output as the flow through the ECMO circuit is in addition to the native cardiac output. The altered hemodynamics with VA-ECMO physiology is the development of the “Harlequin syndrome,” where opposing blood flows from the heart (antegrade, poorly oxygenated blood flow) and the peripheral ECMO cannulation (retrograde, highly oxygenated blood flow) results in lower oxygen levels in the upper body and normal or elevated oxygen levels in the lower body. In contrast, veno-venous ECMO (VV-ECMO) removes deoxygenated blood from a large vein and returns oxygenated blood into the body via a second large vein. Veno-venous ECMO supports oxygenation but is not designed to support circulation (35).

Modifications of the VA-ECMO circuit are often needed to protect the LV and pulmonary circulation. A third configuration often employed in longer-term VA-ECMO is veno-arterial venous-ECMO (VAV-ECMO), often used in patients with differential hypoxia. In VAV-ECMO, an additional cannula is introduced into the jugular (or subclavian) vein to deliver oxygenated blood to the pulmonary circulation. A fourth configuration described in the setting of RV failure is veno-arterial-venous-pulmonary artery-ECMO (VAVPa ECMO). In VAVPa ECMO, a venous catheter is advanced into the pulmonary artery (PA) to provide oxygenated and decarboxylated blood into the systemic and pulmonary circulation. VA ECMO increases LV afterload, which can lead to LV dysfunction**.** With prolonged VA-ECMO, LV unloading can also be achieved percutaneously using several techniques: converting to a VAVPA ECMO configuration or trans-femoral placement of trans-aortic catheter across the aortic valve to drain the LV and drain the LV into the venous arm of the ECMO circuit (38).

## ECMO indications and contraindications

The primary indication for ECMO is the treatment of MPE-induced cardiogenic shock and witnessed cardiac arrest. In cardiogenic shock, the patient should not have known aortic valve incompetence and should not have significant comorbidities such as end-stage heart failure, severe chronic obstructive pulmonary disease (COPD), liver failure, end-stage renal failure (ESRD), or any terminal irreversible illness which would impact longterm survival. In cardiac arrest due to MPE, the patient should be less than 70 years old and have had a witnessed arrest with an interval from cardiopulmonary arrest to first Cardiopulmonary Resuscitation (CPR) of less than 5 min. The observed initial rhythm should be identified as Ventricular Fibrillation (VF), paroxysmal ventricular tachycardia, or pulseless electrical activity, and the patient should not have experienced recurrent VF or intermittent return of spontaneous circulation (39, 40).

The goals of ECMO in MPE are to stabilize the patient by restoring circulation, offloading the right ventricle, and restoring end-organ oxygenation. VA-ECMO provides both pulmonary and cardiac support. It is one of the most reliable and expeditious ways to decrease RV overload, and to improve RV function, thus allowing for hemodynamic stability and restoration of tissue oxygenation. VV-ECMO is used for respiratory support in those unresponsive to mechanical ventilation due to acute, potentially reversible respiratory failure. In this regard, there are three strategies for ECMO utilization in MPE: bridge to definitive therapy, sole therapy, and recovery after treatment. The first strategy provides pre-operative and peri-operative support for open surgical embolectomy or percutaneous thrombectomy. The second strategy is supporting the patient with anticoagulation or systemic thrombolysis without open or percutaneous intervention. The third strategy is to support the patient after intervention to allow for end-organ recovery after the thrombus burden has been removed. Given the significant progress that has been made in the medical response to MPE with improved diagnostic algorithms, the aggressive use of systemic thrombolytics, the rapid deployment of catheter-directed thrombolysis, and percutaneous thrombectomy therapies, ECMO as a bridge to intervention and recovery after treatment has become the commonest utilization reported in the literature.

## Risk stratification

Using predictive algorithms to understand the potential mortality and morbidity of an intervention is a valuable adjunct to clinical decision-making. Despite a high sensitivity and negative predictive value, the Pulmonary Embolism Severity Index (PESI) and simplified Pulmonary Embolism Severity Index (sPESI) lack specificity to predict early mortality because they rely heavily on demographic and co-morbid conditions rather than the severity of the acute PE event. They are not helpful in patients with MPE placed on ECMO. Two predictive scoring systems have been refined to specifically evaluate patients with MPE in whom ECMO is being considered or has been placed emergently—Sequential Organ Failure Assessment-Right Ventricle (SOFA_RV_) and Survival after VA ECMO (SAVE).

The SOFA instrument assesses the extent of organ dysfunction using six different patient criteria.- neurologic, blood, liver, kidney, and blood pressure/hemodynamics and assigns a score within each category (41) (Table 2A). As the SOFA score increases, the likelihood of mortality increases. In the case of ECMO, it is recommended that after a baseline SOFA score is obtained, serial SOFA evaluations are performed over the next 48 h of ECMO, therapy and an increase in SOFA score after 48 h with the presence of hyperlactatemia is significantly associated with subsequent hospital mortality (42). A recent modification of SOFA with the addition of an echocardiographic assessment of the right ventricle has improved the prognostic performance of the original SOFA score in VA-ECMO and is now termed SOFA-Right ventricle (RV)—SOFA_RV_ (43). In the original paper, SOFA_RV_ outperformed the original SAVE in predicting mortality in patients on VA-ECMO. A SOFA_RV_ score less than five is associated with a morality less than 20%, while a score greater than fourteen is associated with a mortality of 95%.

**Table 2A:** Sequential organ failure assessment (SOFA) score.

Variable	Points					
	0	1	2	2	3	4
PaO_2_/FiO_2_, mmHg	≥400	300–399	200–299	≤199 and NOT mechanically ventilated	100–199 and mechanically ventilated	<100 and mechanically ventilated
Platelets, ×10^3^/µl	≥150	100–149	50–99		20–49	<20
Glasgow Coma Scale	15	13–14	10–12		6–9	<6
Bilirubin, mg/dl (μmol/L)	<1.2 (<20)	1.2–1.9 (20–32)	2.0–5.9 (33–101)		6.0–11.9 (102–204)	≥12.0 (>204)
Mean arterial pressure (MAP) OR administration of vasoactive agents required (listed doses are in units of mcg/kg/min)	No hypotension	MAP <70 mmHg	Dopamine ≤5 or Dobutamine (any dose)		Dopamine >5, Epinephrine ≤0.1, or Norepinephrine ≤0.1	Dopamine >15, Epinephrine >0.1, or Norepinephrine >0.1
Creatinine, mg/dl (μmol/L) or urine output (UOP)	<1.2 (<110)	1.2–1.9 (110–170)	2.0–3.4 (171–299)		3.5–4.9 (300–440) or UOP <500 ml/day)	≥5.0 (>440) or UOP <200 ml/day

The SAVE score is a survival prediction score derived from pre-ECMO assessment data extracted from the ELSO registry (44). The original SAVE score utilized age, weight, Central Nervous system (CNS) dysfunction, etiology of the cardiogenic shock, renal function, metabolic acidosis, respiratory and cardiac parameters, and end-organ failure to classify patients into five mortality categories ranging from 18% to 75%. A serum lactate level has been added to the score in more recent iterations to improve prognostic accuracy and demonstrated excellent discrimination when lactate and SAVE scores are combined (45) (Table 2B).

**Table 2B:** Modified survival after VA ECMO (SAVE) score.

			Points
Age (years)	18–38		7
	39–52		4
	53–62		3
	≥63		0
Weight	<143 lbs (<65 kg)		1
	143–196 lbs (65–89 kg)		2
	>196 lbs (>89 kg)		0
Etiology of cardiogenic shock	Myocarditis	No	0
		Yes	3
	Refractory VT/VF	No	0
		Yes	2
	Post heart or lung transplantation	No	0
		Yes	3
	Congenital heart disease	No	0
		Yes	−3
Renal	Acute renal failure	No	0
		Yes	−3
	Chronic renal failure	No	0
		Yes	−6
Metabolic Acidosis	HCO₃ before ECMO ≤15 mmol/L (91.5 mg/dl)	No	0
		Yes	−3
Respiratory	Duration of intubation prior to initiation of ECMO, h	≤10	0
		11–29	−2
		≥30	−4
	Peak inspiratory pressure ≤20 cm H₂O (≤2.0 kPa)	No	0
		Yes	3
Cardiac	Pre-ECMO cardiac arrest	No	0
		Yes	−2
	Diastolic blood pressure within 6 h before ECMO cannulation ≥40 mmHg	No	0
		Yes	3
	Pulse pressure within 6 h before ECMO cannulation ≤20 mmHg	No	0
		Yes	−2
Other organ failures pre-ECMO	Liver failure	No	0
		Yes	−3
	CNS dysfunction	No	0
		Yes	−3
Lactate	<75 mg/dl	No	0
		Yes	15

A SAVE score ≤−10 is associated with a mortality of less than 20%, while a score >5 is associated with a 75% mortality.

A valid criticism of both SOFA_RV_ and SAVE is that they do not provide the practitioner with a tool to decide on the initiation of ECMO but rather an instrument to assist in determining the value of continuation of the ECMO therapy.

## Complications of ECMO

The deployment of ECMO is associated with a significant systemic inflammatory response and a series of complications that can impact the patient's ability to survive and require additional resources to address the issues as they arise (46). Understanding and mitigating these complications enhances immediate patient care and improves early survival.

### Inflammatory complications

The initiation of ECMO leads to the rapid activation of the coagulation cascade, complement systems, endothelial cells, leukocytes, and platelets. Platelet and leukocyte activation then releases multiple proinflammatory cytokines (TNF-α, IL-1, IL-6, IL-8), promotes microcirculatory dysfunction, and induces aseptic parenchymal inflammation and injury in multiple organs. Systemic inflammation and activated coagulation cascade lead to macro- and micro-circulation endothelial activation and dysfunction coupled with microcirculatory thrombosis. Patients on ECMO also develop a dysfunctional gastrointestinal barrier, leading to bacterial translocation and endotoxin release into the bloodstream, which acts as an additional promotor of systemic inflammation.

### Coagulation complications

Bleeding and thrombosis are the most common coagulation complications associated with ECMO. In a systematic review, Sy et al. (47) reported a prevalence of significant bleeding events of 27%, with cannula site and intracranial sites the most commonly reported issues. Bleeding results from the consequences of systemic anticoagulation and ECMO-acquired coagulopathy (thrombocytopenia, platelet dysfunction, Acquired von Willebrand syndrome, hemolysis, and enhanced fibrinolysis). A recent meta-analysis has confirmed that low-dose anticoagulation is a feasible and safe strategy compared to standard-dose anticoagulation in patients supported by ECMO (48). ELSO has issued a guideline on using anticoagulation during ECMO (49).

### Intracranial complications

ECMO-associated brain injury comprises a spectrum from intracranial hemorrhage (ICH), acute ischemic stroke (AIS), new onset seizure activity, cerebral edema, intracranial hypertension, and hypoxic-ischemic encephalopathy (HIE). Neurologic injuries are reported more frequently with VA-ECMO than with VV-ECMO. In a recent systematic review (50), the median incidence of acute neurologic complications was 13%, ranging from 1% to 78%. Of the 13%, 5% are ICH, 5% AIS, 2% are seizures, and 1% are attributed to other causes. The median mortality across 44 studies was 96% for ICH, 84% for AIS, and 40% for new-onset seizure activity. If HIE and brain death were excluded, the median mortality in patients with ECMO-associated brain injury (83%) would be higher than in patients without ECMO (42%).

### Renal complications

Acute kidney injury (AKI) is prevalent in patients placed on ECMO and has been associated with poor outcomes (51). No significant differences in AKI risk have been identified between VV-ECMO and VA-ECMO, but the presence of AKI during VA-ECMO is strongly associated with subsequent mortality (52). More than 75% of the patients placed on ECMO will develop AKI, and the need for dialysis occurs in more than half of these patients. Adult patients who develop severe AKI on ECMO are older, have diabetes mellitus, have higher APACHE II and SOFA scores, and have a prolonged duration of ECMO support (53). The development of AKI on ECMO contributes to the worsening of multi-organ dysfunction due to the accumulation of increased extravascular water that leads to interstitial overload, impairment of oxygen transport in organs, and impairment in pulmonary O_2_ transport.

### Pulmonary complications

VA-ECMO can induce pulmonary injury and congestion related to left ventricle pressure overload. Lung function is adversely affected by parenchymal injury from ECMO-induced systemic inflammatory response, ECMO-induced hemodynamic changes inducing parenchymal ischemia, ECMO-induced lung congestion due to altered ventricular filling, and ischemia-reperfusion injury during ECMO and after decannulation. As a result of these acute changes in VA-ECMO, the lungs develop protein-rich edema, alveolar hemorrhages, tissue necrosis, and fibrosis.

### Limb complications

Acute limb ischemia occurs in 10%–15% of patients on VA ECMO and has been associated with worse outcomes (54–59). Acute limb ischemia is more common in patients who are female, are younger, have pre-existing peripheral vascular disease, and with the use of larger arterial cannulas (>20 Fr) (60–62). The development of acute limb ischemia has been directly associated with the SOFA score calculated at the initiation of ECMO (63). Current guidelines recommend ultrasound-guided access during intial cannulation if percutaneous access is to be used so that the Profunda Femoris artery is identified and its ostium protected, thus preserving collateral flow (64, 65). There is significant variation in the indications for placement of a distal perfusion arterial cannula (DPC), the type of cannula inserted, and the technique of cannula placement (66), Systematic reviews, and meta-analyses have shown that the placement of a functioning DPC can result in an average 16% reduction in the incidence of limb ischemia without a change in overall mortality during VA ECMO (55). The placement of large-size cannulas in the jugular or femoral veins has also been identified as a predisposing factor for deep venous thrombosis (DVT) in those veins (67).

## Outcomes

In 2015, Yusuff and Associates (68) conducted a systematic literature review on ECMO in 78 patients with MPE and reviewed over 20 years of case reports on the topic and found an overall survival of 70.1%. Survival with ECMO was equivalent, irrespective of the adjuvant intervention used to remove pulmonary clots: thrombolysis, catheter-based embolectomy, or surgical embolectomy. Those who had ECMO initiated, while in cardiac arrest, had an overall higher mortality than those who had never experienced such an event. In 2020, Baldetti performed an updated systematic review and pooled analysis of all published experiences of ECMO support in MPE and identified 21 studies with 635 patients. In this combined study population, ECMO was indicated for cardiac arrest in 62.3%, and immediate ECMO support was pursued in 61.9% of patients. 57.0% of patients underwent adjunctive reperfusion therapies. Early all-cause mortality was 41.1%, and in meta-regression analyses, no covariates affected mortality. In 2021, Harwood Scott et al. published a narrower systematic literature review on the outcomes of managing 301 patients experiencing MPE-related cardiac arrest (69). Only Sixty-one percent of patients presenting with cardiac arrest due to MPE survived to discharge. Patients who received systemic thrombolysis for MPE before ECMO cannulation had similar survival compared with patients who had ECMO cannulation without exposure to systemic thrombolysis. There was no significant difference in risk of death if ECMO cannulation occurred in the emergency department or other hospital locations. In an associated multivariate analysis, the authors demonstrated a three-fold increase in the risk of death for patients over 65 years old and a six-fold increase if cannulation occurred during cardiopulmonary resuscitation. A second study in 2021 by Kaso et al. (70) performed a meta-analysis to compare in-hospital mortality in patients treated for MPE with and without ECMO. Eleven eligible studies with 791 patients presenting with MPE were included (270 subjects received ECMO, and 521 subjects did not). The rate of cardiac arrest in this study population was 64% in the ECMO group. Mortality in-hospital was not significantly different between patients treated with and without ECMO. However, these findings were limited by marked study heterogeneity with multiple confounding and selection biases and limited generalizability. There was no evidence of a small study effect. Regarding the decline effect and early-extreme bias, meta-regression demonstrated that publication year was not a significant covariate.

Based on these data sets, it appears that the initiation of ECMO alone, with or without systemic thrombolysis, will not improve outcomes over conventional therapy and that ECMO should be followed by an open or percutaneous thrombectomy to reduce or eliminate the clot burden and rapidly stabilize cardiovascular status.

## Conclusion

The use of ECMO in MPE continues to evolve and has been associated with lower mortality in registry review. Most patients on ECMO also undergo open or interventional PE interventions to treat the MPE. However, there has been no significant difference in outcomes between patients treated with and without ECMO in meta-analyses. Considerable heterogeneity in studies is a significant weakness of the available literature. The application of ECMO in MPE is also associated with substantial multisystem morbidity due to bio-injury, hemorrhagic stroke, renal dysfunction, and bleeding, which must be factored into the outcomes. The application of ECMO in MPE now has a place in current guidelines recommendations but should be combined with an aggressive PE interventional program and strictly adhere to the current selection criteria for ECMO to achieve optimal outcomes.

## References

[1] Martin KA Molsberry R Cuttica MJ Desai KR Schimmel DR Khan SS. Time trends in pulmonary embolism mortality rates in the United States, 1999 to 2018. J Am Heart Assoc. (2020) 9(17):e016784. 10.1161/JAHA.120.016784 32809909 PMC7660782

[2] Becattini C Agnelli G. Predictors of mortality from pulmonary embolism and their influence on clinical management. Thromb Haemost. (2008) 100(05):747–51. 10.1160/TH08-06-0356 18989514

[3] Sakuma M Nakamura M Nakanishi N Miyahara Y Tanabe N Yamada N Inferior vena cava filter is a new additional therapeutic option to reduce mortality from acute pulmonary embolism. Circ J. (2004) 68(9):816–21. 10.1253/circj.68.816 15329501

[4] Kasper W Konstantinides S Geibel A Olschewski M Heinrich F Grosser KD Management strategies and determinants of outcome in acute major pulmonary embolism: results of a multicenter registry. J Am Coll Cardiol. (1997) 30(5):1165–71. 10.1016/s0735-1097(97)00319-7 9350909

[5] Jiménez D Lobo JL Otero R Monreal M Yusen RD. Prognostic significance of multidetector computed tomography in normotensive patients with pulmonary embolism: rationale, methodology and reproducibility for the PROTECT study. J Thromb Thrombolysis. (2012) 34(2):187–92. 10.1007/s11239-012-0709-7 22430281

[6] Pollack CV Schreiber D Goldhaber SZ Slattery D Fanikos J O’Neil BJ Clinical characteristics, management, and outcomes of patients diagnosed with acute pulmonary embolism in the emergency department: initial report of EMPEROR (multicenter emergency medicine pulmonary embolism in the real world registry). J Am Coll Cardiol. (2011) 57(6):700–6. 10.1016/j.jacc.2010.05.071 21292129

[7] Casazza F Becattini C Bongarzoni A Cuccia C Roncon L Favretto G Clinical features and short term outcomes of patients with acute pulmonary embolism. The Italian pulmonary embolism registry (IPER). Thromb Res. (2012) 130:847–52. 10.1016/j.thromres.2012.08.292 22921592

[8] Wood KE. Major pulmonary embolism: review of a pathophysiologic approach to the golden hour of hemodynamically significant pulmonary embolism. Chest. (2002) 121(3):877–905. 10.1378/chest.121.3.877 11888976

[9] Goldberg JB Giri J Kobayashi T Ruel M Mittnacht AJ Rivera-Lebron B Surgical management and mechanical circulatory support in high-risk pulmonary embolisms: historical context, current status, and future directions: a scientific statement from the American heart association. Circulation. (2023) 147(9):e628–47. 10.1161/CIR.0000000000001117 36688837

[10] Maron BA Leopold JA. Emerging concepts in the molecular basis of pulmonary arterial hypertension: part II: neurohormonal signaling contributes to the pulmonary vascular and right ventricular pathophenotype of pulmonary arterial hypertension. Circulation. (2015) 131(23):2079–91. 10.1161/CIRCULATIONAHA.114.006980 26056345 PMC4465126

[11] Prabhakar NR Kumar GK. Mechanisms of sympathetic activation and blood pressure elevation by intermittent hypoxia. Resp Physiol Neurobiol. (2010) 174(1–2):156–61. 10.1016/j.resp.2010.08.021 PMC3042768 20804865

[12] Yu D Wang Y Yu Y Zhong Y Huang L Zhang M Acute beneficial effects of sodium nitroprusside in a rabbit model of massive pulmonary embolism associated with circulatory shock. Am J Path. (2018) 188(8):1768–78. 10.1016/j.ajpath.2018.04.014 29803832

[13] Wang Y Yu D Yu Y Zou W Zeng X Hu L Potential role of sympathetic activity on the pathogenesis of massive pulmonary embolism with circulatory shock in rabbits. Resp Res. (2019) 20:1–10. 10.1186/s12931-019-1069-z PMC6530066 31118045

[14] Dalen JE Haynes FW Hoppin Jr FG Evans GL Bhardwaj P Dexter L. Cardiovascular responses to experimental pulmonary embolism. Am J Cardiol. (1967) 20(1):3–9. 10.1016/0002-9149(67)90104-X

[15] Gurewich V Cohen ML Thomas DP. Humoral factors in massive pulmonary embolism: an experimental study. Am Heart J. (1968) 76(6):784–94. 10.1016/0002-8703(68)90264-0 5721836

[16] Belenkie I Dani R Smith ER Tyberg JV. The importance of pericardial constraint in experimental pulmonary embolism and volume loading. Am Heart J. (1992) 123(3):733–42. 10.1016/0002-8703(92)90514-v 1539525

[17] Zagorski J Kline JA. Differential effect of mild and severe pulmonary embolism on the rat lung transcriptome. Resp Res. (2016) 17(1):1–13. 10.1186/s12931-016-0405-9 PMC4952270 27435598

[18] Watts JA Lee Y-Y Gellar MA Fulkerson M-BK Hwang S-I Kline JA. Proteomics of microparticles after experimental pulmonary embolism. Thromb Res. (2012) 130(1):122–8. 10.1016/j.thromres.2011.09.016 22014850

[19] Cho H-J Kayumov M Kim D Lee K Onyekachi FO Jeung K-W Acute immune response in venoarterial and venovenous extracorporeal membrane oxygenation models of rats. ASAIO J. (2020) 67(5):546–53. 10.1097/MAT.0000000000001265 32826395

[20] Kjærgaard B Kristensen SR Risom M Larsson A. A porcine model of massive, totally occlusive, pulmonary embolism. Thromb Res. (2009) 124(2):226–9. 10.1016/j.thromres.2009.01.010 19232684

[21] Fernandes CJ Assad APL Alves JL Jr Jardim C de Souza R. Pulmonary embolism and gas exchange. Respiration. (2019) 98(3):253–62. 10.1159/000501342 31390642

[22] Riedel M. Acute pulmonary embolism 1: pathophysiology, clinical presentation, and diagnosis. Heart. (2001) 85(2):229–40. 10.1136/heart.85.2.229 11156681 PMC1729607

[23] Smulders YM. Pathophysiology and treatment of haemodynamic instability in acute pulmonary embolism: the pivotal role of pulmonary vasoconstriction. Cardiovasc Res. (2000) 48(1):23–33. 10.1016/s0008-6363(00)00168-1 11033105

[24] Pérez-Nieto OR Gómez-Oropeza I Quintero-Leyra A Kammar-García A Zamarrón-López ÉI Soto-Estrada M Hemodynamic and respiratory support in pulmonary embolism: a narrative review. Front Med. (2023) 10:1123793. 10.3389/fmed.2023.1123793 PMC10272848 37332759

[25] Jaff MR McMurtry MS Archer SL Cushman M Goldenberg N Goldhaber SZ Management of massive and submassive pulmonary embolism, iliofemoral deep vein thrombosis, and chronic thromboembolic pulmonary hypertension: a scientific statement from the American heart association. Circulation. (2011) 123(16):1788–830. 10.1161/CIR.0b013e318214914f 21422387

[26] Konstantinides SV Torbicki A Agnelli G Danchin N Fitzmaurice D Galiè N 2014 ESC guidelines on the diagnosis and management of acute pulmonary embolism. Eur Heart J. (2014) 35(43):3033–69. 10.1093/eurheartj/ehu283 25173341

[27] Konstantinides SV Meyer G Becattini C Bueno H Geersing G-J Harjola V-P 2019 ESC guidelines for the diagnosis and management of acute pulmonary embolism developed in collaboration with the European respiratory society (ERS) the task force for the diagnosis and management of acute pulmonary embolism of the European society of cardiology (ESC). Euro Heart J. (2020) 41(4):543–603. 10.1093/eurheartj/ehz405 31504429

[28] Vanni S Nazerian P Pepe G Baioni M Risso M Grifoni G Comparison of two prognostic models for acute pulmonary embolism: clinical vs. right ventricular dysfunction-guided approach. J Thromb Haemost. (2011) 9(10):1916–23. 10.1111/j.1538-7836.2011.04459.x 21819540

[29] Kearon C Akl EA Comerota AJ Prandoni P Bounameaux H Goldhaber SZ Antithrombotic therapy for VTE disease: antithrombotic therapy and prevention of thrombosis, 9th ed: american college of chest physicians evidence-based clinical practice guidelines. Chest. (2012) 141:e419S–94S. 10.1378/chest.11-2301 22315268 PMC3278049

[30] Leacche M Unic D Goldhaber SZ Rawn JD Aranki SF Couper GS Modern surgical treatment of massive pulmonary embolism: results in 47 consecutive patients after rapid diagnosis and aggressive surgical approach. J Thorac Cardiovasc Surg. (2005) 129(5):1018–23. 10.1016/j.jtcvs.2004.10.023 15867775

[31] Wan S Quinlan DJ Agnelli G Eikelboom JW. Thrombolysis compared with heparin for the initial treatment of pulmonary embolism: a meta-analysis of the randomized controlled trials. Circulation. (2004) 110(6):744–9. 10.1161/01.CIR.0000137826.09715.9C 15262836

[32] Kuo WT Gould MK Louie JD Rosenberg JK Sze DY Hofmann LV. Catheter-directed therapy for the treatment of massive pulmonary embolism: systematic review and meta-analysis of modern techniques. J Vasc Intervent Radiol. (2009) 20(11):1431–40. 10.1016/j.jvir.2009.08.002 19875060

[33] Giri J Sista AK Weinberg I Kearon C Kumbhani DJ Desai ND Interventional therapies for acute pulmonary embolism: current status and principles for the development of novel evidence: a scientific statement from the American heart association. Circulation. (2019) 140(20):e774–801. 10.1161/CIR.0000000000000707 31585051

[34] Lorusso R Shekar K MacLaren G Schmidt M Pellegrino V Meyns B ELSO interim guidelines for venoarterial extracorporeal membrane oxygenation in adult cardiac patients. ASAIO J. (2021) 67(8):827–44. 10.1097/MAT.0000000000001510 34339398

[35] Mid Carolina Internal Medicine Association. An Introduction to Extracorporeal Membrane Oxygenation (ECMO) (2004). Available at: http://www.perfusion.com/cgi-bin/absolutenm/templates/articledisplay.asp?articleid=1807#.VHicmodM7Uw

[36] Hill JD O'Brien TG Murray JJ Dontigny L Bramson M Osborn J Prolonged extracorporeal oxygenation for acute post-traumatic respiratory failure (shock-lung syndrome) use of the bramson membrane lung. N Engl J Med. (1972) 286(12):629–34. 10.1056/NEJM197203232861204 5060491

[37] Davies MJ Arsiwala SS Moore HM Kerr S Sosnowski AW Firmin RK. Extracorporeal membrane oxygenation for the treatment of massive pulmonary embolism. Ann Thorac Surg. (1995) 60(6):1801–3. 10.1016/0003-4975(95)00622-2 8787489

[38] Brasseur A Scolletta S Lorusso R Taccone FS. Hybrid extracorporeal membrane oxygenation. J Thorac Dis. (2018) 10(Suppl. 5):S707. 10.21037/jtd.2018.03.84 29732190 PMC5911562

[39] George N Stephens K Ball E Crandall C Ouchi K Unruh M Extracorporeal membrane oxygenation for cardiac arrest: does age matter? Crit Care Med. (2023) 10:1097. 10.1097/CCM.0000000000006039 PMC11267242 37782526

[40] Richardson ASC Tonna JE Nanjayya V Nixon P Abrams DC Raman L Extracorporeal cardiopulmonary resuscitation in adults. Interim guideline consensus statement from the extracorporeal life support organization. ASAIO J. (2021) 67(3):221. 10.1097/MAT.0000000000001344 33627592 PMC7984716

[41] Minne L Abu-Hanna A de Jonge E. Evaluation of SOFA-based models for predicting mortality in the ICU: a systematic review. Crit Care. (2008) 12(6):1–13. 10.1186/cc7160 PMC2646326 19091120

[42] Laimoud M Alanazi M. The validity of SOFA score to predict mortality in adult patients with cardiogenic shock on venoarterial extracorporeal membrane oxygenation. Crit Care Res Pract. (2020) 2020:3129864. 10.1155/2020/3129864 32963830 PMC7495164

[43] Akin S Caliskan K Soliman O Muslem R Guven G van Thiel RJ A novel mortality risk score predicting intensive care mortality in cardiogenic shock patients treated with veno-arterial extracorporeal membrane oxygenation. J Critical Care. (2020) 55:35–41. 10.1016/j.jcrc.2019.09.017 31689611

[44] Schmidt M Burrell A Roberts L Bailey M Sheldrake J Rycus PT Predicting survival after ECMO for refractory cardiogenic shock: the survival after veno-arterial-ECMO (SAVE)-score. Euro Heart J. (2015) 36(33):2246–56. 10.1093/eurheartj/ehv194 26033984

[45] Chen W-C Huang K-Y Yao C-W Wu C-F Liang S-J Li C-H The modified SAVE score: predicting survival using urgent veno-arterial extracorporeal membrane oxygenation within 24 h of arrival at the emergency department. Critical Care. (2016) 20:1–7. 10.1186/s13054-016-1520-1 27769308 PMC5075192

[46] Aubron C Cheng AC Pilcher D Leong T Magrin G Cooper DJ Factors associated with outcomes of patients on extracorporeal membrane oxygenation support: a 5-year cohort study. Crit Care. (2013) 17:1–12. 10.1186/cc12681 PMC4056036 23594433

[47] Sy E Sklar MC Lequier L Fan E Kanji HD. Anticoagulation practices and the prevalence of major bleeding, thromboembolic events, and mortality in venoarterial extracorporeal membrane oxygenation: a systematic review and meta-analysis. J Crit Care. (2017) 39:87–96. 10.1016/j.jcrc.2017.02.014 28237895

[48] Lv X Deng M Wang L Dong Y Chen L Dai X. Low vs standardized dose anticoagulation regimens for extracorporeal membrane oxygenation: a meta-analysis. PLoS One. (2021) 16(4):e0249854. 10.1371/journal.pone.0249854 33831104 PMC8031334

[49] McMichael AB Ryerson LM Ratano D Fan E Faraoni D Annich GM. 2021 ELSO adult and pediatric anticoagulation guidelines. ASAIO J. (2021) 68(3):303–10. 10.1097/MAT.0000000000001652 35080509

[50] Sutter R Tisljar K Marsch S. Acute neurologic complications during extracorporeal membrane oxygenation: a systematic review. Crit Care Med. (2018) 46(9):1506–13. 10.1097/CCM.0000000000003223 29782356

[51] Chen Y-C Tsai F-C Fang J-T Yang C-W. Acute kidney injury in adults receiving extracorporeal membrane oxygenation. J Formosan Med Assoc. (2014) 113(11):778–85. 10.1016/j.jfma.2014.04.006 24928419

[52] Mou Z He J Guan T Chen L. Acute kidney injury during extracorporeal membrane oxygenation: VA ECMO versus VV ECMO. J Intens Care Med. (2022) 37(6):743–52. 10.1177/08850666211035323 34397300

[53] Mou Z Guan T Chen L. Risk factors of acute kidney injury in ECMO patients: a systematic review and meta-analysis. J Intens Care Med. (2022) 37(2):267–77. 10.1177/08850666211003485 33761767

[54] Wong JK Melvin AL Joshi DJ Lee CY Archibald WJ Angona RE Cannulation-related complications on veno-arterial extracorporeal membrane oxygenation: prevalence and effect on mortality. Artif Organ. (2017) 41(9):827–34. 10.1111/aor.12880 28589655

[55] Jia D Yang IX Ling RR Syn N Poon WH Murughan K Vascular complications of extracorporeal membrane oxygenation: a systematic review and meta-regression analysis. Crit Care Med. (2020) 48(12):e1269–77. 10.1097/CCM.0000000000004688 33105148

[56] Tanaka D Hirose H Cavarocchi N Entwistle JW. The impact of vascular complications on survival of patients on venoarterial extracorporeal membrane oxygenation. Ann Thorac Surg. (2016) 101(5):1729–34. 10.1016/j.athoracsur.2015.10.095 26872734

[57] Roussel A Al-Attar N Alkhoder S Radu C Raffoul R Alshammari M Outcomes of percutaneous femoral cannulation for venoarterial extracorporeal membrane oxygenation support. Euro Heart J: Acute Cardiovasc Care. (2012) 1(2):111–4. 10.1177/2048872612449417 PMC3760531 24062897

[58] Mosquera VX Solla-Buceta M Pradas-Irún C Fernández-Arias L. Lower limb overflow syndrome in extracorporeal membrane oxygenation. Interact Cardiovasc Thorac Surg. (2014) 19(3):532–4. 10.1093/icvts/ivu165 24899594

[59] Steffen RJ Sale S Anandamurthy B Cruz VB Grady PM Soltesz EG Using near-infrared spectroscopy to monitor lower extremities in patients on venoarterial extracorporeal membrane oxygenation. Ann Thorac Surg. (2014) 98(5):1853–4. 10.1016/j.athoracsur.2014.04.057 25441810

[60] Gulkarov I Khusid E Worku B Demissie S Guerges M Salemi A Meta-analysis of the effect of vascular complications on mortality in patients undergoing femoral venoarterial extracorporeal membrane oxygenation. Ann Vasc Surg. (2021) 71:488–95. 10.1016/j.avsg.2020.09.042 33160061

[61] Bonicolini E Martucci G Simons J Raffa GM Spina C Lo Coco V Limb ischemia in peripheral veno-arterial extracorporeal membrane oxygenation: a narrative review of incidence, prevention, monitoring, and treatment. Crit Care. (2019) 23(1):1–17. 10.1186/s13054-019-2541-3 31362770 PMC6668078

[62] Kim J Cho YH Sung K Park TK Lee GY Lee JM Impact of cannula size on clinical outcomes in peripheral venoarterial extracorporeal membrane oxygenation. ASAIO J. (2019) 65(6):573–9. 10.1097/MAT.0000000000000858 30074498

[63] Danial P Hajage D Nguyen LS Mastroianni C Demondion P Schmidt M Percutaneous versus surgical femoro-femoral veno-arterial ECMO: a propensity score matched study. Intens Care Med. (2018) 44:2153–61. 10.1007/s00134-018-5442-z 30430207

[64] Rupp S Apfelbaum J Blitt C Caplan R Connis R Domino K. American society of anesthesiologists task force on central venous AccessPractice guidelines for central venous access: a report by the American society of anesthesiologists task force on central venous access. Anesthesiology. (2012) 116(3):539–73. 10.1097/ALN.0b013e31823c9569 22307320

[65] Lamperti M Bodenham AR Pittiruti M Blaivas M Augoustides JG Elbarbary M International evidence-based recommendations on ultrasound-guided vascular access. Intens Care Med. (2012) 38:1105–17. 10.1007/s00134-012-2597-x 22614241

[66] Juo YY Skancke M Sanaiha Y Mantha A Jimenez JC Benharash P. Efficacy of distal perfusion cannulae in preventing limb ischemia during extracorporeal membrane oxygenation: a systematic review and meta-analysis. Artif Organs. (2017) 41(11):E263–73. 10.1111/aor.12942 28762511

[67] Ma RWL Huilgol RL Granger E Jackson A Saling S Dower A Does a distal perfusion cannula reduce ischaemic complications of extracorporeal membrane oxygenation? ANZ J Surg. (2016) 86(12):1002–6. 10.1111/ans.13441 26923903

[68] Yusuff H Zochios V Vuylsteke A. Extracorporeal membrane oxygenation in acute massive pulmonary embolism: a systematic review. Perfusion. (2015) 30(8):611–6. 10.1177/0267659115583377 25910837

[69] Scott JH Gordon M Vender R Pettigrew S Desai P Marchetti N Venoarterial extracorporeal membrane oxygenation in massive pulmonary embolism-related cardiac arrest: a systematic review. Crit Care Med. (2021) 49(5):760–9. 10.1097/CCM.0000000000004828 33590996

[70] Kaso ER Pan JA Salerno M Kadl A Aldridge C Haskal ZJ Venoarterial extracorporeal membrane oxygenation for acute massive pulmonary embolism: a meta-analysis and call to action. J Cardiovasc Trans Res. (2022) 15:258–67. 10.1007/s12265-021-10158-0 PMC8288068 34282541

